# 9-Year Follow-Up of a Pediatric LQTS Patient With a Subcutaneous ICD: Considerations for ILR Monitoring

**DOI:** 10.1016/j.jaccas.2025.104568

**Published:** 2025-08-06

**Authors:** Yuya Nakamura, Yoshikazu Suzuki, Haruka Miyazaki, Taku Asano, Youichi Kobayashi, Toshiro Shinke

**Affiliations:** Division of Cardiology, Department of Medicine, Showa University School of Medicine, Tokyo, Japan

**Keywords:** implantable loop recorder, long QT syndrome, pediatric, subcutaneous implantable cardioverter-defibrillator, torsades de pointes

## Abstract

**Background:**

Long QT syndrome (LQTS) is a congenital arrhythmic disorder associated with sudden cardiac death. In patients at low to intermediate risk, implantable loop recorders (ILRs) are sometimes used for arrhythmic surveillance. Subcutaneous implantable cardioverter-defibrillators (S-ICDs) are considered in children, but long-term follow-up data remain limited.

**Case Summary:**

A 9-year-old patient with genetically confirmed LQT1 initially received an ILR. After a syncopal event, an S-ICD was implanted using a 2-incision intermuscular and C-curve technique to accommodate anticipated growth. The S-ICD recorded a short episode of torsades de pointes (TdP) that was not detected by the ILR. After reprogramming with more permissive settings, both devices successfully detected subsequent TdP episodes. Over 9 years of follow-up, the S-ICD maintained stable sensing and function.

**Discussion:**

This case illustrates the diagnostic limitations of ILRs and demonstrates the feasibility and durability of growth-conscious S-ICD implantation in pediatric patients with LQTS.

## Graphical Abstract

**Figure:**
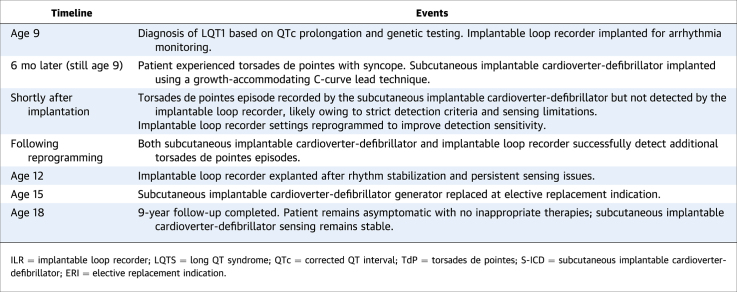

## History of presentation

A 9-year-old boy was referred to our hospital with suspected long QT syndrome (LQTS). His mother and older brother had been diagnosed with LQT1. An initial electrocardiogram revealed sinus rhythm with a heart rate of 58 beats/min, a PQ interval of 120 ms, a QRS duration of 82 ms, and a QTc of 588 ms (Bazett's formula).

## Diagnostic assessment

**Figure 1:**
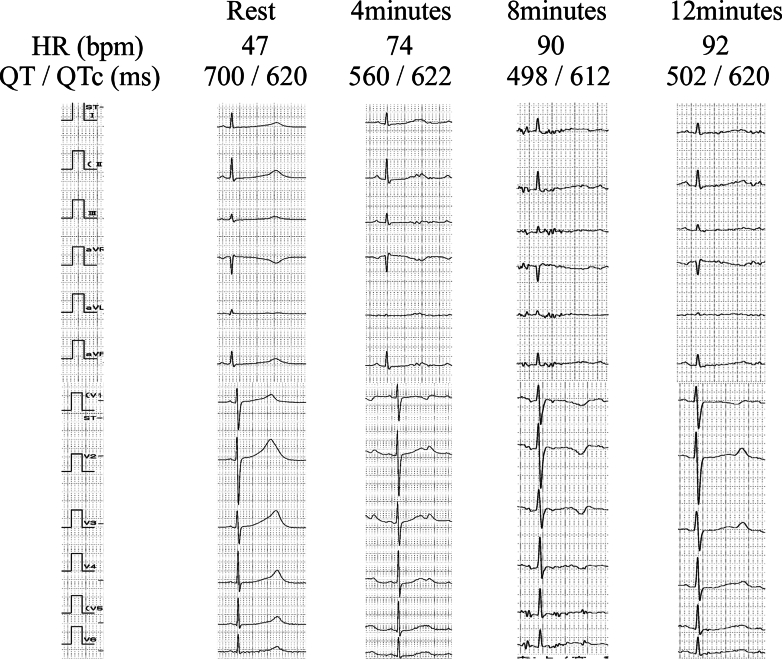
QTc Dynamics During Exercise Testing Surface electrocardiograms recorded at baseline and at 4, 8, and 12 minutes into a 25-W exercise stress test. Heart rate (HR), QT interval, and QTc (Bazett’s formula) are displayed for each time point. Although the absolute QT interval shortened with increasing heart rate, the QTc remained prolonged throughout. The T waves showed transient inversion followed by normalization.

As he had no history of syncope at the initial visit, an exercise stress test was conducted, which demonstrated QTc prolongation up to 622 ms (Bazett's formula) (Figure 1). Genetic testing confirmed LQT1 with a KCNQ1 mutation (c.938T>A, c.939C>A, p.I313K).

## Management and intervention

The patient was initially managed with metoprolol (80 mg/d) and exercise restrictions. Following his first syncopal episode, implantation of a transvenous implantable cardioverter-defibrillator (TV-ICD) was recommended as an urgent measure. However, his family strongly preferred to wait until the subcutaneous implantable cardioverter-defibrillator (S-ICD), EMBLEM S-ICD (Boston Scientific), became available in Japan and declined TV-ICD implantation in consideration of their child's future growth and lifestyle. Therefore, an implantable loop recorder (ILR), Reveal XT (Medtronic), was implanted to monitor for potential arrhythmic events, and exercise restrictions were again strongly emphasized.

The S-ICD became available 6 months after ILR implantation, and the family agreed to proceed with the procedure. The S-ICD implantation was chosen owing to the patient's age, lack of pacing indication, and the family's preference to avoid a transvenous system.^1^ Preoperative screening was conducted in both standing and supine positions as well as during an exercise stress test, assuming both parallel and C-curve lead configurations. It was confirmed that all 3 sensing vectors were compatible and that the QRS morphology was not significantly affected by posture or physical activity. His height was 135.6 cm and weight was 28.6 kg at 9 years of age. Given the anticipated growth of more than 35 cm (father’s height: 170 cm; mother’s height: 158 cm), a 2-incision intermuscular technique and C-curve implantation was selected. During lead placement, we deliberately curved the tunneler to form a smooth C-curve, leaving slack in the shock lead to accommodate future growth. Ultrasonography was used to locate the latissimus dorsi–serratus anterior boundary, positioning the generator posterior to the latissimus dorsi–serratus anterior interface. The procedure was successfully completed under general anesthesia without complications. Defibrillation threshold testing was not performed at the time of initial S-ICD implantation owing to the absence of sustained ventricular fibrillation (VF); however, the system impedance was 43 Ω, which was within the appropriate range. The conditional and shock zones were programmed at 200 to 250 beats/min and >250 beats/min, respectively.

**Figure 2:**
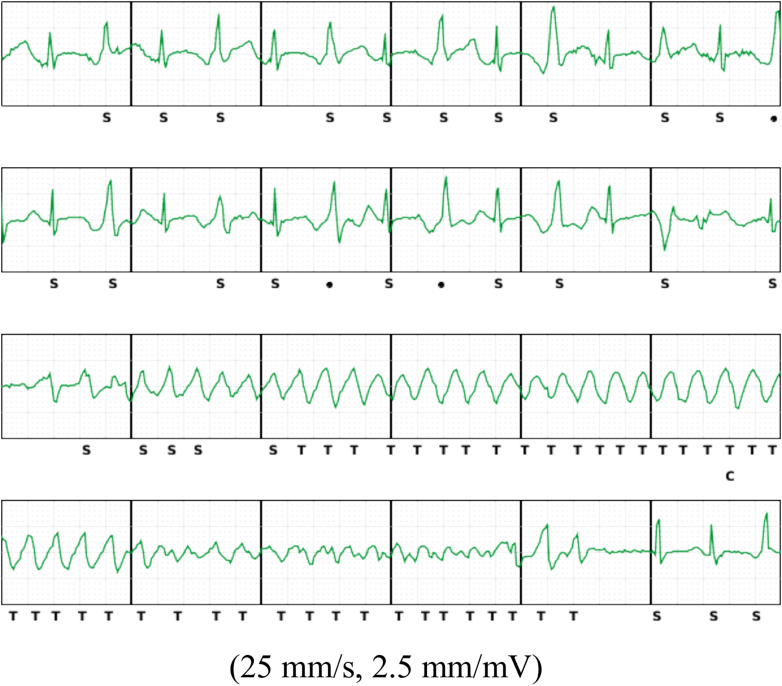
S-ICD Electrogram of a TdP Episode Subcutaneous implantable cardioverter-defibrillator (S-ICD) electrogram showing a 10-second episode of torsades de pointes (TdP) recorded at 25 mm/s and 2.5 mm/mV. This episode was not detected by the implantable loop recorder, likely owing to restrictive initial detection criteria.

**Figure 3:**
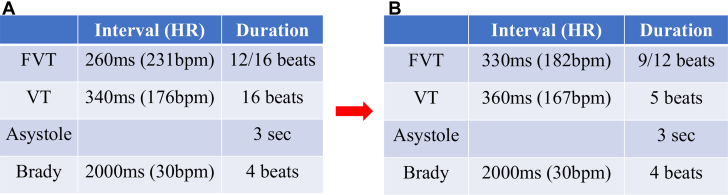
Implantable Loop Recorder Detection Settings Before and After Reprogramming (A) Initial settings: Fast ventricular tachycardia (FVT) was detected at 260 ms (231 beats/min), requiring 12 of 16 intervals; ventricular tachycardia (VT) was detected at 340 ms (176 beats/min), requiring 16 consecutive intervals. (B) After reprogramming: FVT detection was adjusted to 330 ms (182 beats/min), requiring 9 of 12 intervals; VT detection was adjusted to 360 ms (167 beats/min), requiring 5 consecutive intervals. These changes aimed to improve torsades de pointes detection sensitivity. Brady = bradycardia.

**Figure 4:**
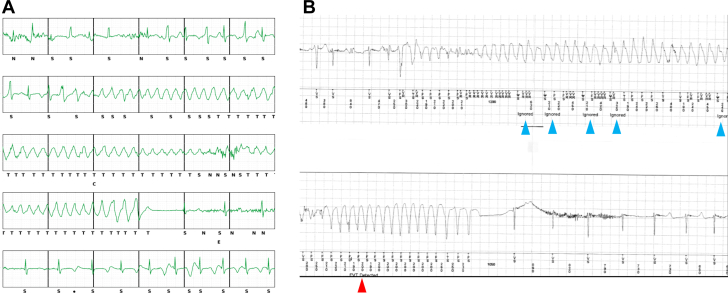
S-ICD and ILR Electrograms After ILR Reprogramming Electrograms of the same torsades de pointes episode from the subcutaneous implantable cardioverter-defibrillator (S-ICD) (A) and implantable loop recorder (ILR) (B) after reprogramming. The ILR tracing (B) shows sensing abnormalities, including episodes of undersensing and refractory period–related ignored counts (blue arrowheads), which likely reduced the ventricular tachycardia/fast ventricular tachycardia detection counters. This episode was not classified as ventricular tachycardia owing to ignored counts, but was ultimately detected as fast ventricular tachycardia (red arrowhead). Waveform analysis suggests that similar anomalies may have contributed to previous detection failures.

After S-ICD implantation, the patient experienced another syncopal episode at 10 years of age. The S-ICD successfully recorded a short episode of torsades de pointes (TdP), whereas the ILR failed to detect the arrhythmia (Figure 2). Review of the ILR settings revealed that strict detection criteria had been applied to avoid oversensing of sinus tachycardia, which may have inadvertently led to underdetection of TdP. To enhance sensitivity, the ILR parameters for fast ventricular tachycardia (FVT) and ventricular tachycardia (VT) were reprogrammed with more lenient thresholds and reduced detection counts (Figures 3A and 3B). Following reprogramming, 4 additional TdP episodes were successfully detected by both the ILR and the S-ICD (Figures 4A and 4B). The ILR was explanted at age 12 because of battery depletion. At 15 years of age, 6 years after the initial S-ICD implantation, generator replacement was performed. The generator was replaced owing to elective replacement indication. During the procedure, the fibrous capsule around the generator was removed. Defibrillation threshold testing confirmed adequate safety margins, successfully terminating VF with a single 65 J shock. The shock impedance was measured at 65 Ω. No complications, such as pocket hematoma or infection, were observed.

## Postoperative follow-up

**Figure 5:**
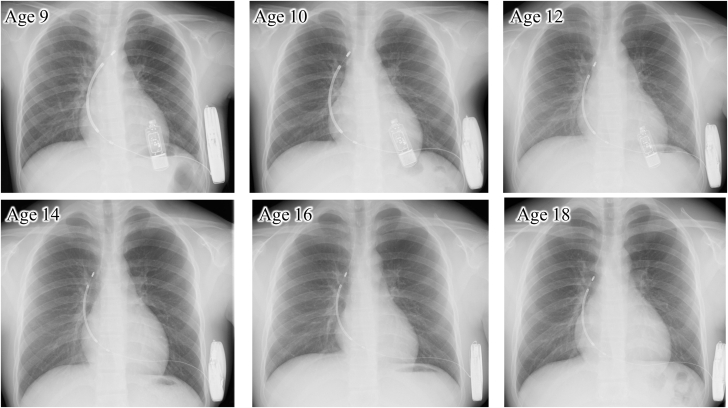
Serial Chest Radiographs During Growth Chest radiographs obtained at ages 9, 10, 12, 14, 16, and 18 years demonstrate stable positioning of the subcutaneous implantable cardioverter-defibrillator lead over time. The C-curve lead placement with intentional slack successfully accommodated a height increase of more than 35 cm during growth.

Despite significant growth over the years (height 173 cm at age 18), the S-ICD lead remained stable 9 years after initial implantation, with both the primary and the secondary sensing vectors remaining appropriate. Proper arrhythmia detection was maintained, and no inappropriate shocks or T-wave oversensing occurred throughout the follow-up period. Device interrogation was performed every 6 months via in-clinic and remote monitoring. Serial chest radiographs (ages 9-18) confirmed stable S-ICD lead positioning throughout the growth period (Figure 5).

## Discussion

This case highlights 2 key considerations for pediatric LQTS. First, short episodes of TdP may not be detected by ILRs owing to limited detection algorithms and memory capacity. This underscores the need to reprogram ILR settings individually for pediatric patients with LQTS. Second, growth must be considered when implanting an ICD or S-ICD. In this case, a 2-incision S-ICD implanted with a C-curve lead maintained stable positioning over 9 years of follow-up.

### Challenges of ILR in detecting short-duration TdP episodes

ILR implantation may be considered as a temporary measure for some low- to intermediate-risk patients.^2^^,^^3^ However, in pediatric cases, strict exercise restrictions may not always be adhered to, and if tachycardia detection criteria are too lenient, sinus tachycardia can occupy the device's memory, potentially overwriting true arrhythmic events. On the other hand, when the primary concern is TdP, there is a potential risk of underdiagnosing short-duration episodes owing to their brevity, especially when using strict detection criteria, for the following reasons^4^:

1. Certain R waves may occur within the postsensing blanking period or the subsequent refractory period and are therefore not sensed. As a result, the device may fail to accumulate enough detections to meet the criteria for a VT or FVT episode. Moreover, repeated sensing during the refractory period may reduce the detection counter as these beats are marked as ignored.
2. Some R waves during the TdP episode may have amplitudes below the autoadjusting sensing threshold, resulting in undersensing. During polymorphic arrhythmias such as TdP, the variability in signal amplitude may prevent consistent detection of all ventricular activations.
3. The morphology of the R waves during TdP may be unclear, and the noise rejection algorithm may be activated after preliminary detection of an FVT episode resulting in clearing of the detection counters.
4. The irregularity and prolongation of RR intervals during TdP may prevent consistent accumulation of detection counts, either by failing to meet VT/FVT rate thresholds or by disrupting the continuity required by the algorithm.

Because of the above-listed factors, the episode may terminate spontaneously before meeting the detection criteria for a VT or FVT episode. As shown in this case, strict detection criteria may contribute to underdiagnosis of short TdP episodes. ILRs are not substitutes for ICDs or S-ICDs in high-risk pediatric patients with LQTS. In lower-risk patients, optimizing ILR settings is crucial to avoid underdetection of TdP while limiting oversensing. Newer ILRs offer enhanced storage, daily remote monitoring, and active symptom recording, which may improve detection of true TdP events.

### Growth considerations in S-ICD implantation for pediatric LQTS

This case further highlights the critical importance of growth considerations in pediatric patients. Given the anticipated height increase of approximately 35 cm, based on parental stature, the S-ICD was implanted with this expected growth in mind. TV-ICDs are often considered in pediatric patients owing to their pacing capabilities; however, in this case, an S-ICD was selected to avoid lead-related complications and ensure long-term system stability.

Recent data from a European multicenter registry (SIDECAR) support the safety and efficacy of S-ICD therapy in pediatric and young adult patients with inherited arrhythmia syndromes and structural heart disease. Appropriate and inappropriate shock rates were 17% and 13%, respectively, with no defibrillation failures, confirming its viability in selected young patients.^5^ These findings are consistent with our experience and reinforce the suitability of S-ICD implantation in pediatric patients with LQTS without pacing indication.

In this case, the 2-incision technique and C-curve lead placement allowed for natural slack, optimal vector sensing, and stable system impedance, maintaining appropriate function even into adulthood.

## Limitations

This case presents a unique example of a pediatric patient with LQTS who underwent S-ICD implantation and was followed for 9 years, providing valuable insights into long-term device stability and effectiveness. However, certain limitations should be acknowledged. As the generator becomes encapsulated over time, there is a potential for increased shock impedance and defibrillation thresholds over the ultra-long term. To ensure the safe use of S-ICDs in pediatric patients, it is essential to evaluate these long-term device management issues. Further accumulation of cases with extended follow-up will be necessary to assess the safety and performance of S-ICDs in children over decades.

## Conclusions

This case demonstrates the considerations of ILRs in detecting brief episodes of TdP in pediatric patients with LQTS and emphasizes the importance of optimizing detection parameters to avoid underdiagnosis. S-ICDs can provide reliable long-term arrhythmic protection when implanted with careful consideration of growth. This case highlights the limitations of ILRs in detecting short TdP episodes and the durability of S-ICD therapy when growth is considered. To our knowledge, this is the first report to provide radiographic and clinical evidence of stable S-ICD performance over 9 years in a pediatric patient with LQTS. Continued accumulation of long-term follow-up cases is essential to further establish the safety and efficacy of S-ICDs in pediatric populations.

**Visual Summary:** Timeline of Clinical Events

Timeline	Events
Age 9	Diagnosis of LQT1 based on QTc prolongation and genetic testing. Implantable loop recorder implanted for arrhythmia monitoring.
6 mo later (still age 9)	Patient experienced torsades de pointes with syncope. Subcutaneous implantable cardioverter-defibrillator implanted using a growth-accommodating C-curve lead technique.
Shortly after implantation	Torsades de pointes episode recorded by the subcutaneous implantable cardioverter-defibrillator but not detected by the implantable loop recorder, likely owing to strict detection criteria and sensing limitations. Implantable loop recorder settings reprogrammed to improve detection sensitivity.
Following reprogramming	Both subcutaneous implantable cardioverter-defibrillator and implantable loop recorder successfully detect additional torsades de pointes episodes.
Age 12	Implantable loop recorder explanted after rhythm stabilization and persistent sensing issues.
Age 15	Subcutaneous implantable cardioverter-defibrillator generator replaced at elective replacement indication.
Age 18	9-year follow-up completed. Patient remains asymptomatic with no inappropriate therapies; subcutaneous implantable cardioverter-defibrillator sensing remains stable.

ILR = implantable loop recorder; LQTS = long QT syndrome; QTc = corrected QT interval; TdP = torsades de pointes; S-ICD = subcutaneous implantable cardioverter-defibrillator; ERI = elective replacement indication.

**Equipment List:** Device and Monitoring Equipment Used in This Case

• Implantable Loop Recorder (ILR): Reveal XT (Medtronic Japan) • Used for arrhythmia monitoring from age 9 to 12 • Reprogrammed at age 10 to improve detection of torsades de pointes (TdP) • Subcutaneous Implantable Cardioverter-Defibrillator (S-ICD): EMBLEM S-ICD (Boston Scientific Japan) • Implanted at age 9 using a C-curve lead placement technique to accommodate growth • Generator replaced at age 15 due to elective replacement indication (ERI)

## Funding Support and Author Disclosures

The authors have reported that they have no relationships relevant to the contents of this paper to disclose.

**Take-Home Messages**

• ILRs may fail to detect short-duration TdP episodes unless detection settings are carefully optimized.
• S-ICD implantation strategies that take growth into consideration can ensure stable long-term function in pediatric patients.
